# In Memoriam – Dr. James Kirkpatrick

**DOI:** 10.24908/pocusj.v10i01.19258

**Published:** 2025-04-15

**Authors:** Amer M. Johri, Nova Panebianco, Benjamin Galen

**Affiliations:** 1 Division of Cardiology, Department of Medicine, Queen's University, Kingston, CAN; 2 Department of Emergency Medicine, Perelman School of Medicine, University of Pennsylvania, Philadelphia, PA, USA; 3 Department of Medicine, Albert Einstein College of Medicine and Montefiore Medical Center, Bronx, NY, USA

**Keywords:** remarks, in memoriam

**Figure:**
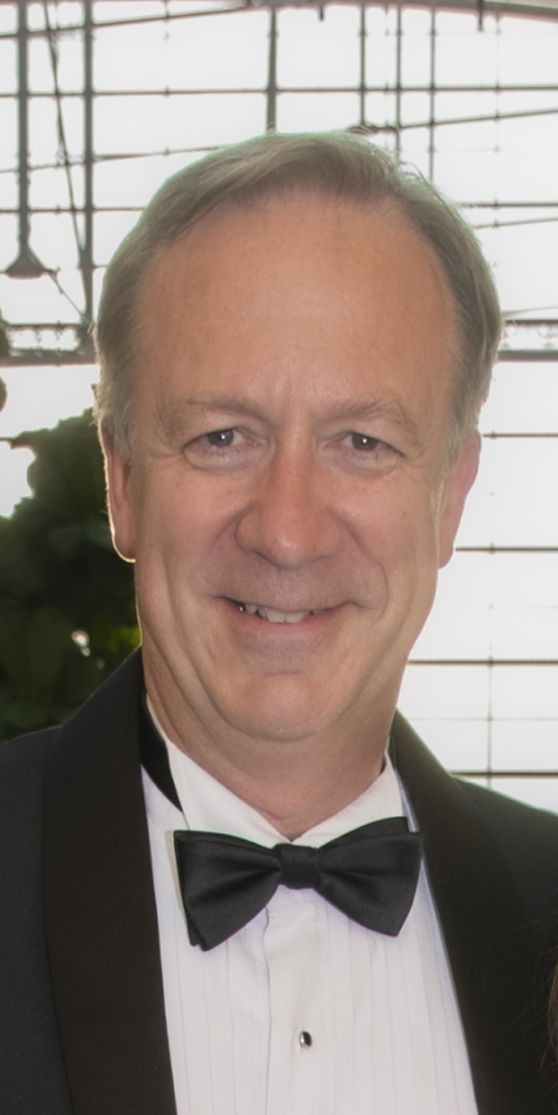


It is with great sadness that we remark upon the passing of Dr. James Kirkpatrick in this issue of the POCUS Journal. Jim was widely admired and respected in the POCUS community for his enthusiasm towards point of care imaging and his embrace of learners and colleagues from around the world.

Jim had an incredible way of giving to and mentoring those around him- a quiet magnetism that drew people in, supported them, and helped them achieve their goals. But Jim was also a force of nature- with his booming baritone and confident leadership. We all looked up to his strength and vision during uncertain times such as the pandemic, or to navigate complex topics and debates to achieve consensus.

Some of his many contributions related to POCUS included a guideline to implement ultrasound during the pandemic [1], developing nomenclature for POCUS [2], and of course contributing to our Journal [3]. These activities allowed him to impact many different disciplines including cardiology, anesthesia, critical care, and emergency medicine, thus impacting a significant part of our POCUS community.

From the mountain of Jim's work there will always run a clear stream of thought- a source of knowledge we can always draw inspiration from during personal challenges and while solving problems together. Jim would ask his POCUS colleagues to remember him not for his awards and success, but for his empathy and compassion towards his colleagues. He would ask us to continue the work – using POCUS to bring knowledge, technology, and innovation to those in need and to inspire others to create more solutions to health problems our world faces. He was passionate about POCUS for this reason, and his example reminds us of the opportunity we have in our unique community to make the world better and improve health through technology, innovation, and the embrace of multiple points of view. We are so lucky to have had Jim in our lives and to benefit from the great vision he leaves us.
